# Probable Stevens–Johnson Syndrome During Taletrectinib Treatment After Crizotinib‐Related Rash in ROS1‐Positive Lung Cancer

**DOI:** 10.1002/rcr2.70782

**Published:** 2026-10-29

**Authors:** Hirotoshi Yasui, Ryo Fukaura, Miyu Misaki, Souma Usui, Saho Katsumata, Ryo Niimi, Ko Ohara, Kaito Sano, Yasutaka Mori, Tomofumi Shibata, Yasutaka Fukui, Mitsuru Odate, Yasushi Makino

**Affiliations:** ^1^ Department of Respiratory Medicine Toyohashi Municipal Hospital Toyohashi Aichi Japan; ^2^ Department of Dermatology Toyohashi Municipal Hospital Toyohashi Aichi Japan

**Keywords:** Crizotinib, ROS1‐positive lung cancer, severe cutaneous adverse reaction, Stevens–Johnson syndrome, taletrectinib

## Abstract

Severe cutaneous adverse reactions to taletrectinib, a next‐generation ROS1 inhibitor for ROS1‐rearranged non‐small cell lung cancer, remain poorly described. A 52‐year‐old woman with ROS1‐rearranged lung adenocarcinoma developed a Grade 3 rash during crizotinib therapy. After improvement, taletrectinib was initiated. On Day 21, conjunctival hyperaemia appeared, followed by high fever, neutropenia, widespread trunk‐dominant erythema, and lower‐lip mucosal involvement. Although epidermal detachment, a positive Nikolsky sign, and characteristic epidermal necrosis were absent, and typical target lesions were not documented during the acute phase, the clinical course was considered consistent with probable Stevens–Johnson syndrome. Fever and ocular symptoms rapidly improved after taletrectinib discontinuation and systemic corticosteroid therapy. Infection and human herpesvirus 6 (HHV‐6) reactivation were not identified, and ALDEN and Naranjo assessments supported a probable association with taletrectinib. During ROS1‐TKI therapy, fever and neutropenia should prompt consideration of severe cutaneous adverse reactions as well as infection.

## Graphical abstract

Probable Stevens–Johnson syndrome developed during taletrectinib treatment after a preceding crizotinib‐related Grade 3 rash in a patient with ROS1‐rearranged lung adenocarcinoma. This case highlights that severe cutaneous adverse reactions should be considered when fever and neutropenia occur during ROS1‐TKI therapy.

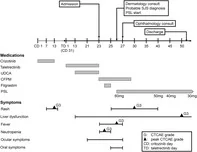

## 1 Introduction

ROS1 rearrangements are actionable driver alterations in non‐small cell lung cancer and are targetable with ROS1 tyrosine kinase inhibitors (TKIs). Taletrectinib is a ROS1‐TKI, but severe cutaneous adverse reactions remain poorly described [1]. SJS/TEN has been reported with targeted anticancer therapies, including TKIs, requiring early recognition and prompt drug withdrawal [2, 3]. Herein, we report probable SJS during taletrectinib treatment after a Grade 3 crizotinib‐related rash, with fever and neutropenia preceding overt cutaneous manifestations.

## 2 Case Report

A 52‐year‐old never‐smoking woman with performance status 0 was diagnosed with right lower lobe lung adenocarcinoma (cT2aN2M1c, stage IVB) with brain metastases. Multigene testing revealed a ROS1 rearrangement, and the PD‐L1 tumour proportion score was 100%. After stereotactic irradiation, crizotinib was initiated as first‐line therapy (crizotinib Day 1: CD1).

A rash appeared on CD7 and progressed to widespread Grade 3 erythema on CD13; crizotinib was discontinued. After improvement, taletrectinib was initiated on CD31 (taletrectinib Day 1: TD1). Figure 1 summarises the clinical course.

**FIGURE 1:**
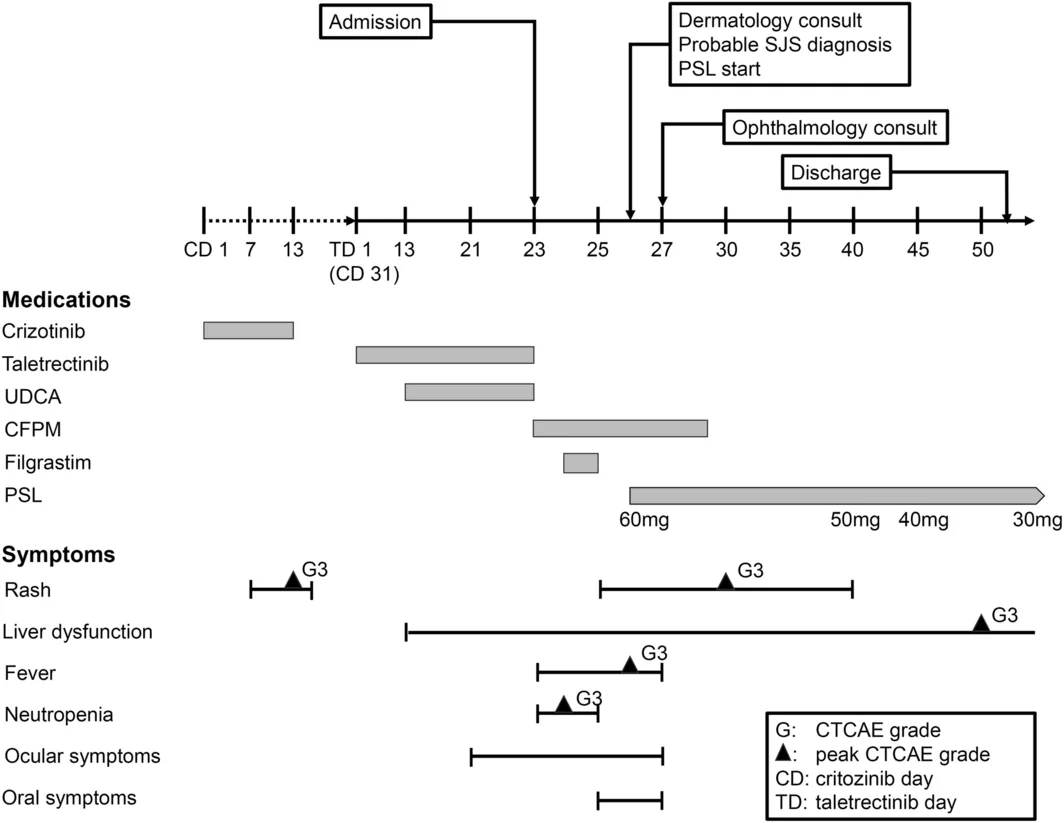
Clinical course after crizotinib and taletrectinib treatment. Crizotinib was discontinued because of Grade 3 rash. After the rash improved, taletrectinib was initiated, followed by ocular symptoms, fever, neutropenia, and widespread erythematous rash with lip mucosal involvement. The patient was clinically diagnosed with probable Stevens–Johnson syndrome, and prednisolone was started on TD26. Triangles indicate the peak CTCAE grade for each adverse event. CD, crizotinib days; CFPM, cefepime; G, CTCAE grade; PSL, prednisolone; TD, taletrectinib days; UDCA, ursodeoxycholic acid.

On TD13, mild AST and ALT elevations developed, and ursodeoxycholic acid was initiated. On TD21, conjunctival hyperaemia and eye discharge appeared. On TD23, she presented with fever > 39°C, leucopenia (1790/μL), and neutropenia (700/μL) and was admitted for suspected febrile neutropenia. Conjunctival hyperaemia was present, with no apparent skin rash or oral mucosal lesions. Procalcitonin, blood cultures, COVID‐19 and influenza testing, and contrast‐enhanced CT showed no evidence of infection. All medications, including taletrectinib, were discontinued, and cefepime was initiated. Fever of 40°C persisted the next day with worsening neutropenia (631/μL); filgrastim was administered.

On TD26, leucocyte and neutrophil counts improved, but high fever persisted and an erythematous rash became evident. Dermatological examination showed trunk‐dominant erythema, lower‐lip mucosal involvement, and preceding ocular symptoms. Typical target lesions were not documented, and probable SJS was clinically diagnosed. Skin biopsy on TD26 showed perivascular dermatitis with histiocytic and lymphocytic infiltration without characteristic full‐thickness epidermal necrosis (Figure 2A–C). HHV‐6 DNA, EBV DNA, and CMV‐C7‐HRP were negative on diagnostic and pre–third‐line therapy testing.

**FIGURE 2:**
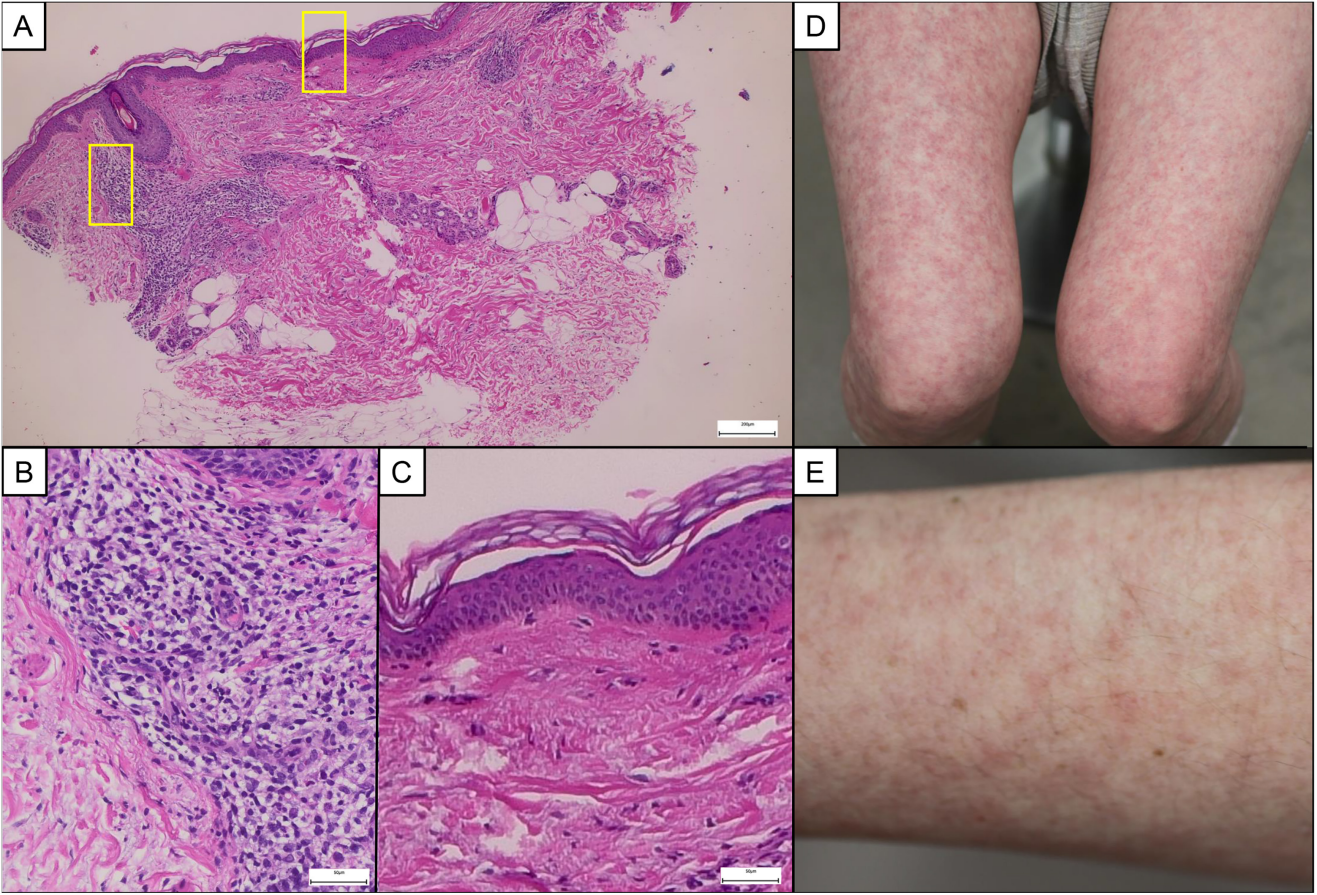
Histopathological and clinical findings of the cutaneous eruption. (A–C) Skin biopsy performed on TD26, shortly after the cutaneous eruption became clinically apparent. (A) Low‐magnification view showing perivascular inflammatory cell infiltration in the dermis. (B) Higher‐magnification view showing predominantly histiocytic and lymphocytic infiltration around dermal vessels. (C) Higher‐magnification view showing preserved epidermis and dermo‐epidermal junction without characteristic full‐thickness epidermal necrosis (haematoxylin and eosin staining; scale bars, 200, 50 and 50 μm, respectively). (D, E) Clinical photographs obtained approximately one week after initiation of systemic corticosteroid therapy (TD34). (D) Overview of the proximal lower extremities showing residual diffuse erythematous macules and patches. (E) Close‐up view of the upper arm showing erythematous lesions, some with targetoid morphology.

Prednisolone 60 mg/day was started on TD26. Conjunctival hyperaemia improved by TD27 and fever resolved by TD28. Ophthalmological examination showed no corneal damage or apparent keratoconjunctival epithelial involvement. On TD30, confluent erythema involving the trunk, upper arms, and thighs reached 50% of body surface area without epidermal detachment or a positive Nikolsky sign. The lip lesion resolved, and the rash evolved to pigmentation with desquamation. Photographs obtained approximately 1 week after corticosteroid initiation showed residual proximal‐extremity erythema, some with targetoid morphology (Figure 2D,E). ALT peaked at 501 U/L on TD48 before improving.

Drug‐induced lymphocyte stimulation tests were negative for ursodeoxycholic acid, crizotinib, and taletrectinib. ALDEN and Naranjo scores were 5 and 7, respectively, indicating a probable association with taletrectinib. Cefepime was unlikely to have initiated the reaction because ocular symptoms and fever preceded its administration. Ursodeoxycholic acid, started 8 days before ocular symptoms, could not be completely excluded. Taletrectinib was considered the most likely causative drug based on chronology and causality assessments. Prednisolone was tapered and discontinued on TD131; third‐line carboplatin, pemetrexed, and bevacizumab were initiated on TD124. Imaging before third‐line therapy showed that the partial response achieved during taletrectinib treatment was maintained.

## 3 Discussion

This case describes probable SJS during taletrectinib treatment after a Grade 3 crizotinib‐related rash. SJS/TEN has been reported with targeted anticancer therapies, including TKIs [2]. In the FDA multidisciplinary review, one treatment‐related serious event was reported as SJS among 337 patients with ROS1‐positive NSCLC receiving taletrectinib 600 mg once daily; however, the FDA later assessed the event as more consistent with DRESS than SJS [1]. This case is notable for sequential cutaneous reactions to two ROS1‐TKIs and for ocular symptoms, fever, and neutropenia preceding overt cutaneous manifestations.

Diagnostic certainty was limited by the absence of epidermal detachment, a positive Nikolsky sign, characteristic epidermal necrosis, and documented typical target lesions during the acute phase. Nevertheless, high fever, conjunctival and lip mucosal involvement, widespread trunk‐dominant erythema followed by desquamation, compatible drug chronology and no identified infection supported probable SJS or an SJS‐spectrum severe cutaneous adverse reaction [3]. The biopsy was obtained shortly after the eruption became apparent, which may have contributed to the absence of fully developed histopathological findings. Erythema multiforme major remained an important differential diagnosis and could not be completely excluded. Photographs obtained approximately 1 week after corticosteroid initiation showed targetoid proximal‐extremity lesions, but their diagnostic significance was limited because they were obtained after treatment and the initial eruption was predominantly truncal [3].

The sequential reactions raise the possibility of individual susceptibility to TKI‐associated cutaneous hypersensitivity but do not establish immunological cross‐reactivity. This remains hypothetical because mechanistic evidence was lacking and drug‐induced lymphocyte stimulation tests were negative.

ALDEN and Naranjo scores supported taletrectinib as the most likely causative agent [4]. Cefepime was less likely because ocular symptoms and fever preceded its administration, whereas ursodeoxycholic acid could not be completely excluded. Fever and neutropenia initially mimicked febrile neutropenia, but infection was not documented and fever resolved after taletrectinib discontinuation and corticosteroid treatment. Neutropenia can occur in SJS/TEN and has been associated with clinical outcomes [5]. Therefore, fever and neutropenia during TKI therapy should prompt consideration of severe cutaneous adverse reactions alongside infection. Untreated acute‐phase photographs, including mucosal lesions, were unavailable.

Overall, probable SJS should be considered when fever, neutropenia, and mucocutaneous symptoms develop during taletrectinib therapy. Early withdrawal of the suspected drug and prompt specialist evaluation are essential.

## Author Contributions

H.Y. conceptualised the case report and drafted the original manuscript. H.Y., R.F., and M.M. contributed to the clinical data acquisition and investigation. S.U., S.K., R.N., K.O., K.S., Y.Mo., T.S., Y.F., M.O., and Y.Ma. contributed to manuscript review and editing. Y.Ma. supervised the study.

## Funding

The authors have nothing to report.

## Ethics Statement

The authors have nothing to report.

## Consent

The authors declare that written informed consent was obtained from the patient for the publication of this manuscript and the accompanying figures using the consent form provided by the journal.

## Conflicts of Interest

Toyohashi Municipal Hospital received payments from Nippon Kayaku Co. Ltd. for an adverse event report related to taletrectinib. The authors declare no conflicts of interest.

## Data Availability

The data that support the findings of this study are available on request from the corresponding author. The data are not publicly available due to privacy or ethical restrictions.
